# The variation of antenatal corticosteroids administration for the singleton preterm birth in China, 2017 to 2018

**DOI:** 10.1186/s12887-022-03529-2

**Published:** 2022-08-03

**Authors:** Qing Wang, Siyuan Jiang, Xuefeng Hu, Chao Chen, Yun Cao, Shoo Kim Lee, Jiang-Qin Liu, On behalf of the Reduction of Infection in Neonatal Intensive Care Units using the Evidence-based Practice for Improving Quality (REIN-EPIQ) Study Group, Yong Ji, Shuping Han, Sannan Wang, Zhankui Li, Shiwen Xia, Changyi Yang, Chuanzhong Yang, Ling Chen, Jing Yuan, Ling Liu, Bin Yi, Zhenlang Lin, Yang Wang, Ling He, Mingxia Li, Xinnian Pan, Yan Guo, Cuiqing Liu, Qin Zhou, Xiaoying Li, Hong Xiong, Yujie Qi, Mingyan Hei

**Affiliations:** 1 Department of Neonatology, Shanghai First Maternity and Infant Hospital, Tongji University School of Medicine, #2699, Gaoke western Road, Pudong District, Shanghai, 201204 China; 2 Department of Neonatology, Children’s Hospital of Fudan University, #399, Wanyuan Road, Minghang District, Shanghai, 201102 China; 3 Maternal-Infant Care Research Centre and Department of Pediatrics Mount Sinai Hospital, Department of Pediatrics, and #Department of Obstetrics and Gynecology and Dalla Lana School of Public Health, University of Toronto, 600 University Avenue, Room 19-231M, Toronto, ON M5G 1X5 Canada

**Keywords:** Antenatal corticosteroids, Preterm, Perinatal factors, Neonatal mortality, Bronchopulmonary dysplasia

## Abstract

**Background:**

The administration of antenatal corticosteroids (ACS) to women who are at risk of preterm birth has been proven to reduce not only the mortality, but also the major morbidities of the preterm infants. The rate of ACS and the risk factors associated with ACS use in Chinese population is unclear. This study aimed to investigate the rate of ACS use and the associated perinatal factors in the tertiary maternal centers of China.

**Methods:**

Data for this retrospective observational study came from a clinical database of preterm infants established by REIN-EPIQ trial. All infants born at < 34 weeks of gestation and admitted to 18 tertiary maternal centers in China from 2017 to 2018 were enrolled. Any dose of dexamethasone was given prior to preterm delivery was recorded and the associated perinatal factors were analyzed.

**Results:**

The rate of ACS exposure in this population was 71.2% (range 20.2 – 92%) and the ACS use in these 18 maternal centers varied from 20.2 to 92.0% in this period. ACS exposure was higher among women with preeclampsia, caesarean section delivery, antibiotic treatment and who delivered infants with lower gestational age and small for gestational age. ACS use was highest in the 28–31 weeks gestational age group, and lowest in the under 26 weeks of gestational age group (x^2^ = 65.478, *P* < 0.001). ACS exposure was associated with lower odds of bronchopulmonary dysplasia or death (OR, 0.778; 95% CI 0.661 to 0.916) and invasive respiration requirement (OR, 0.668; 95% CI 0.585 to 0.762) in this population.

**Conclusion:**

The ACS exposure was variable among maternity hospitals and quality improvement of ACS administration is warranted.

## Backgrounds

Preterm birth has been increasing in China in recent decades, and accounted for 6.9% of live birth or 1.1 million preterm infants annually in 2019 [1]. Preterm birth is a leading cause of neonatal mortality in China, second only to perinatal asphyxia [2]. Consequently, management of preterm birth and improvement of preterm birth outcomes is a priority for China.

Administration of antenatal corticosteroids (ACS) to women who are at risk of preterm birth has been proven to decrease the mortality of preterm infants and reduce not only major morbidities like neonatal respiratory distress syndrome (NRDS), necrotizing enterocolitis (NEC) and intraventricular hemorrhage (IVH), but also improve long term developmental outcomes [3]. ACS has been widely accepted as standard of care for anticipated preterm deliveries between 24 to 34 weeks of gestational age [4, 5]. The best timing of the ACS is within 7 days of and prior to premature delivery [6]. One repeat course is recommended for pregnant women below 34 weeks of gestational age who have received one prior course of ACS for risk of preterm delivery and more than 2 repeated courses of ACS are not recommended [7]. ACS is safe for pregnant women [8]. Nevertheless, the prevalence of ACS administration varies in different gestational ages and different maternal centers and is reported to be between 70–90% among pregnant women less than 34 weeks of gestational age in high income countries [9, 10] and 50–53% in China [11, 12]. This gap merits investigation and needs to be reduced to improve the care of preterm infants. In this study, we aim to analyze the any use of ACS among tertiary level maternity and infant health centers in China, to gain insights that may facilitate development of a strategy of quality improvement to increase the ACS use rate.

## Methods

### Overview

Data for this retrospective observational study came from a clinical database of preterm infants established by REIN-EPIQ (REduction of Infection in Neonatal intensive care units using the Evidence-based Practice for Improving Quality) trial (REIN-EPIQ study, clinicaltrials.gov #NCT02600195) [13]. The study was reviewed by the ethics committee of Children’s Hospital of Fudan University. The consent from the parents was waivered by the ethical committee regarding to the retrospective data abstraction from each hospital. REIN-EPIQ collected standardized maternal and infant data from 25 tertiary level neonatal intensive care units (NICU), including 18 maternity hospitals and 7 children’s hospitals from May 2015 to April 2018 for the purpose of quality improvement for managing infection and antibiotic use in level III NICUs in China.

### Population

The subjects were preterm infants whose gestational age was less than 34 weeks. The inclusion criteria for the study were: (1) gestational age < 34 weeks, Gestational age was determined using the hierarchy of best obstetric estimate based on prenatal ultrasound, menstrual history or obstetric examination; or (2) birth weight < 1500 g; (3) admission to the NICU of member hospitals of REIN-EPIQ within 7 days of birth; (4) discharge time from May 1, 2015 to April 30, 2018. Exclusion criteria were: children with congenital malformations, including severe organ structural malformations and chromosomal abnormalities.

A total of 27,534 children were included in the REIN-EPIQ database during this period. Only inborn preterm infants were included in this study because there was a high possibility of missing perinatal data among outborn infants. All infants from 7 children’s hospitals were also excluded due to the possibility of duplication of record. We excluded data prior to 2017 because twins were not identified prior to that time.

**Fig. 1:**
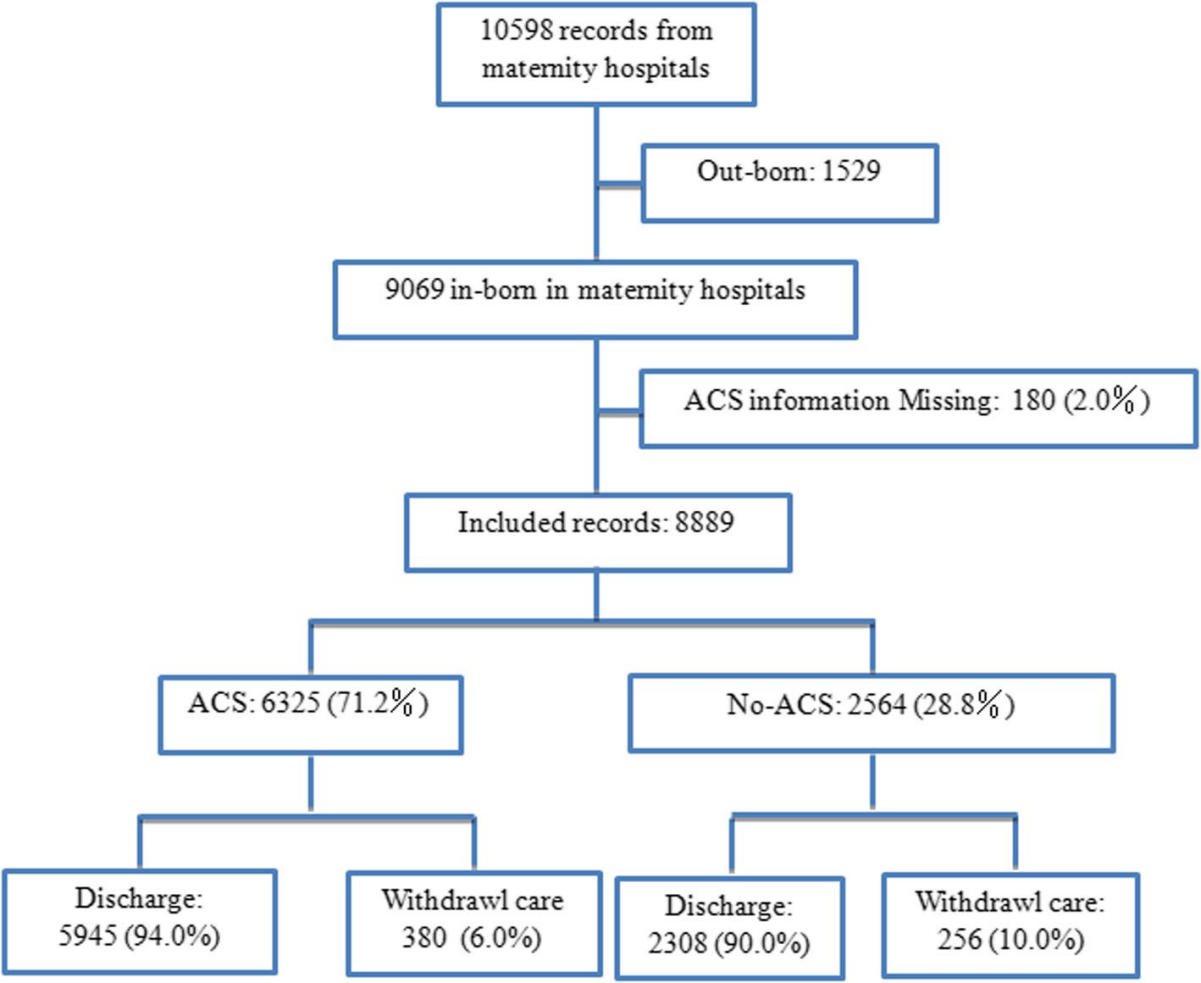
Flow diagram of research cohort. ACS: antenatal corticosteroids

During 2017 and 2018, there were 10,598 singleton preterm infants below 34 weeks of GA admitted into the 18 participating maternity hospital NICUs. We excluded 1529 out-born infants as well as 180 in-born infants with missing information on ACS use. The remaining 8,889 infants were included in the analysis. Of these, 636 infants were discharged against medical advice. (Fig. 1).

### Data collection and data quality control

A standardized database was used for data collection, including maternal and infant baseline data, and information on clinical care and outcomes of infants. A trained and dedicated data abstractor collected data at each site using standardized data definitions established prior to study commencement. Data were uploaded monthly to the research center of Children’s Hospital of Fudan University, and data integrity and quality were checked by the research center.

### Measures and definitions

ACS administration was defined as any dose of dexamethasone administration prior to preterm delivery. Whether the ACS course was complete or incomplete was not identified in this database. There is a systemic registration of pregnancy by the government to manage the risk of pregnancy over the country. If the women register into the system and receive regular checks during the pregnancy. It is defined as “regular antenatal care”. If the pregnant women didn’t register and visit the obstetricians regually until birth. It was defined as “no regular antenatal care” in this database. Hypertensive disorders of pregnancy (HDP) was defined as either chronic hypertension (persistent elevation of blood pressure before 20 weeks of gestation or prior to pregnancy) or pregnancy-induced hypertension if blood pressure > 145/95 was first recorded after 20 weeks of gestation. Prelabor rupture of membranes (PROM) was defined as membrane rupture more than 6 h before the onset of regular spontaneous uterine contractions. Prenatal antibiotics was defined as administration of antibiotics during second and third trimester of pregnancy. The Transport Risk Index of Physiologic Stability (TRIPS) score was used as an illness severity score on NICU admission [14]. Bronchopulmonary dysplasia (BPD) was defined as mechanical ventilation or oxygen dependency at 36 weeks of postmenstrual age or discharge [15].

### Statistical analysis

Stata / SE 15.0 software was used for statistical analysis. For normally distributed data, Mean ± SD, and t-test were used for comparison between groups; for non-normally distributed data, Median (Q1, Q3), and rank sum test were used instead. Count data were expressed as frequency and rate, and the χ 2 test or Fisher exact probability method were used for comparison between groups. Logistic multiple regression analysis was used to analyze for risk factors associated with ACS. The *P* < 0.05 level of significance was used.

## Results

The prevalence of ACS use (at least one dose) was 71.2% (6325/8889). On univariate analysis, women who received ACS prior to delivery were more likely to have regular antenatal care, HDP, PROM, prenatal antibiotics, and delivery by cesarean section (CS) compared to those with no ACS exposure (Table 1). Infants exposed to ACS during pregnancy had smaller birth weight and gestational age, and were more likely to be SGA and have less Apgar score < 4 at 1 and 5 min of life.

**Table 1:** Univariate analysis of perinatal factors

Variables	ACS n(%)	No ACS n(%)	*P* value
n	6325	2564	
Primigravida	1794/6321(28.4)	744/2564(29.0)	0.548
Regular prenatal care	6262/6317(99.1)	2486/2546(97.6)	< 0.001
Preclampsia	1344/6306(21.3)	379/2542(14.9)	< 0.001
GDM	898/6306(14.2)	354/2544(13.4)	0.691
PROM > 18 h	2138/6238(34.3)	459/2514(18.3)	< 0.001
Maternal Antibiotics	2421/5775(41.9)	469/2374(19.8)	< 0.001
Cesarean section	3402/5943(57.2)	1201/2308(52.0)	< 0.001
Gestational age (week)	31.2 ± 1.9	31.4 ± 2.1	< 0.001
Birth weight (gram)	1608 ± 404	1686 ± 443	< 0.001
Male	3562/6325(56.3)	1472/2564(57.4)	0.346
SGA	895/6325(14.2)	297/2564(11.6)	0.001
1’ Apgar ≤ 3	244/6316(3.86)	164/2553(6.42)	< 0.001
5’ Apgar ≤ 3	54/6209(0.87)	33/2451(1.35)	0.045
TRIPS (6300/2558)	12.5 ± 10.0	13.2 ± 11.1	0.006
Death or BPD	1427/12184(11.7)	588/4861(12.1)	0.483
Death	492/12184(4.0)	263/4861(5.4)	< 0.001
BPD	1053/11058(9.5)	396/4442(8.9)	0.240
Invasive Ventilation	2709/12184(22.2)	1231/4861(25.3)	< 0.001

*ACS* Antenatal corticosteroids, *GDM* Gestational diabetes mellitus, *PROM* Prelabor rupture of membranes, *SGA* Small for gestational age, *TRIPS* Transport risk index of physiologic stability, *BPD* Bronchopulmonary dysplasia

Logistic regression analysis of perinatal factors showed that factors independently associated with ACS exposure were small for gestational age (SGA), HDP, CS, PROM and prenatal antibiotics. There was no correlation between the use of ACS and infant gender, GDM and primipara (Table 2). Multivariate logistic regression also showed that infants exposed to ACS during pregnancy had smaller birth weight and gestational age, and were more likely to be SGA and have less Apgar score < 4 at 1 and 5 min of life (Table 1). ACS exposure was associated with lower odds of BPD or death (OR, 0.778; 95% CI 0.661 to 0.916), death (OR, 0.608; 95% CI 0.478 to 0.774), BPD (OR, 0.806; 95% CI 0.679 to 0.955) and invasive respiration requirement (OR, 0.668; 95% CI 0.585 to 0.762).

**Table 2:** Logistic regression of perinatal factors associated with antenatal corticosteroids administration

Variables	OR	95% CI		*P* value
Gestational age	0.911	0.890	0.933	< 0.001
small for gestational age	1.241	1.083	1.423	0.002
Male	0.936	0.858	1.021	0.138
Preeclampsia	1.628	1.432	1.851	< 0.001
gestational diabetes mellitus	1.069	0.945	1.209	0.292
Primigravida	0.978	0.894	1.071	0.633
Cesarean section	1.347	1.224	1.482	< 0.001
premature rupture of membranes	1.414	1.243	1.609	< 0.001
Antibiotics	2.604	2.309	2.936	< 0.001

When stratified by gestational age, the proportion of exposed ACS in infants less than 26 weeks, 26–27 weeks, 28–31 weeks and 32–33 weeks gestational age were 54.1%, 65.6%, 74.8% and 68.2% respectively. ACS use was highest in the 28–31 weeks gestational age group, and lowest in the under 26 weeks gestational age group (x^2^ = 65.478, *P* < 0.001). The incidence of ACS exposure was 70.8% among infants with BW less than 1000 g, 75.2% among infants with BW between 1000 and 1499 g, 71.5% among infants with BW between 1500 and 1999 g, and 63.7% among infants with BW greater than or equal to 2000 g. ACS exposure was highest among infants with BW 1000-1499 g, and lowest among infants with BW less than 1000 g (x^2^ = 71.196, *P* < 0.001).

**Fig. 2:**
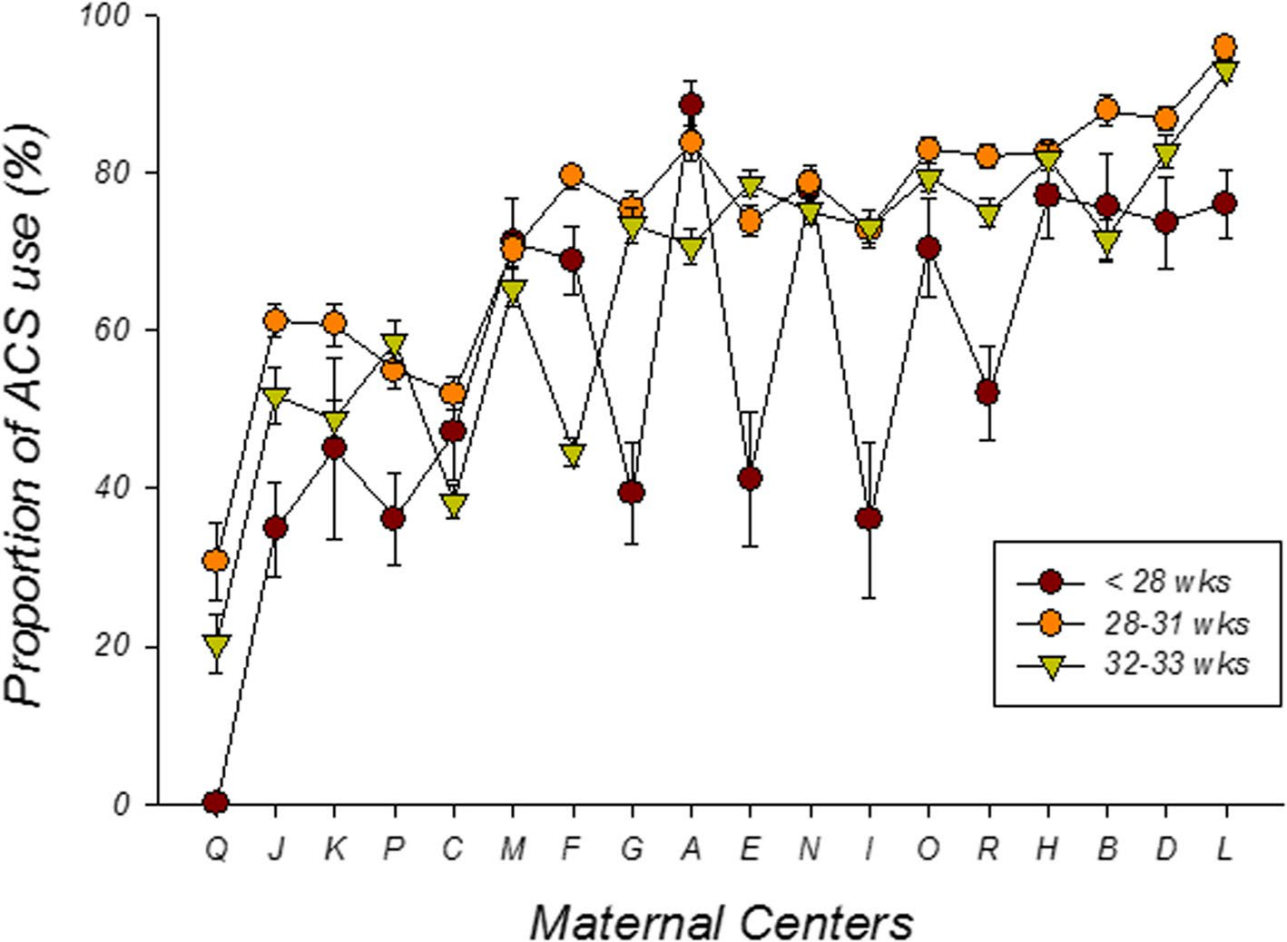
Proportion of antenatal corticosteroids (ACS, y axis) in different maternal centers (x axis) on preterm infants of less than 28 weeks (dark red), 28–31 weeks (orange) and 32–33 weeks of gestational age (green)

The proportion of ACS use varied from 20.2 to 92.0% in these 18 maternal centers. There was significant inter-institutional variation in ACS use for different gestational age groups (Fig. 2). The proportion of ACS use was positively correlated with the number of the infants (Pearson coefficient 0.487, *p* = 0.04), and particularly so among very low birth weight infants (Pearson’s coefficient 0.524, *p* = 0.03). The ACS use was not correlated with the per-capita disposable income on year of 2017–2018 of the city where the maternal center is in (pearson’s coefficient 0.022, *p* = 0.93).

## Discussion

Antenatal corticosteroids administration has become an important obstetrical practice for improving the outcomes of preterm infants less than 34 weeks of gestational age since 1972 [8]. It reduces the risk of neonatal mortality and morbidities including IVH, NEC, and ROP in every gestational age group [10]. ACS use in North America and Europe were reported to be between 70–91.4% [20, 21], which is significantly higher than the 50–56% reported previously in China [13]. Although the 71.2% incidence reported in our study is a significant improvement over previous reports, there is still room for improvement in China.

Administration of ACS to pregnant women at risk of preterm delivery is standard of care for obstetricians in China. Usually a course of intra-muscular dexamethasone (6 mg at 12 h interval for two days) is used in China instead of the betamethasone (12 mg at 24 h of interval for two days) used in North America and Europe [22]. Brownfoot et al. reported that dexamethasone may be associated with lower incidence of IVH and shorter duration of hospitalization but the data is inconclusive [22]. A more recent study reported no significant difference in outcomes at 2 years of age [23]. Dexamethasone and betamethasone are both safe for pregnant women [23]. Although infants previously exposed to these ACS have an increased risk of long-term adverse neurodevelopmental and neurosensory outcomes when delivered at term [24], there was no evidence that a single course of ACS increased the risk of metabolic disease long term [25]. However, there may be risks in repeated courses of ACS [26, 27]. It is very challenging for obstetricians to accurately predict whether preterm delivery will occur within one week and when ACS should be optimally administered [28]. Existing tests for predicting preterm birth are inaccurate and can result in missed opportunities for using ACS [29, 30]. In a Japanese report, there was a high chance of missing the ACS for pregnant women who received tocolysis due to the risk of preterm delivery while only 23% were given ACS [31]. In our study, the women who had preterm related complications were more likely to receive ACS, including small for gestational age, preeclampsia and PROM. We extrapolated that these maternal complications increase the awareness of use ACS on obstetricians. However, recognizing the risk factors associated with missing ACS would be more valuable to the next step of this quality improvement project and it was less investigated in the literature. Therefore, developing a standardized protocol of ACS administration to the women at risk of preterm delivery and investigating the reason of missing ACS will improve not only the use of ACS but also the timing of ACS exposure in this population.

### Variation of ACS among maternity hospitals in China

Understanding the reasons for missing ACS in pregnant women less than 34 weeks GA is very important for quality improvement [32]. Regional variations in incidence of ACS administration present an opportunity for improvement. For example, inter-institutional ACS use varied from 23 to 76% with an average of 58% in Canada in 1996–1997 [33]. Following a national quality improvement effort, this improved to 91.4% and inter-institutional variation was significantly reduced [34]. Outcomes of these infants were also significantly improved [21]. Many perinatal collaboratives have worked on quality improvement of ACS administration by focusing on reducing missed opportunities and optimizing the appropriate time of use [35]. By establishing a reliable practice culture, Kaplan et al. reported that ACS use increased from 76% at baseline to 86% [36]. Similarly, in a report from California from 2005 to 2011, ACS use was increased from 82 to 87.9% with a quality improvement strategy. They also found that a lower level of care was associated with lower incidence of ACS use[37]. Of significance, the ACS use is lower in low and middle income countries, where the majority of preterm death occur [38]. According to the *Every Newborn* *Action Plan* report, the use of ACS varies from 4 to 74% among low and middle income countries [39]. Therefore, reducing regional differences is a viable strategy for improving ACS use and outcomes of preterm infants.

It is of noted in our investigation that the ACS use varies very large over the country in China, from 20.2 to 92.0% in these 18 maternal centers. This variation can be narrowed by the similar quality improvement strategies [36, 37]. Obviously, the number of the preterm infants admitted in a maternal center is positively related with the ACS rate in this study. We also noticed that the preterm infants of less than 28 weeks of GA were exposed to ACS much less than those of above 28 wks in most maternal hospitals (15/18, Fig. 2). There is increasing evidence that exposure to ACS was associated with a lower risk of mortality in extremely preterm infants [40, 41]. Delivery this information to the obstetricians will be one of the important knowledge in the framework of our quality improvement strategy.

### Limitations

This is a retrospective study and only singleton births were included. Information on complete versus incomplete course, or multiple courses of ACS was not available. The time of the ACS administration was also not available in this database. These information are extremely important for the quality improvement purpose in the future. The knowledge level of obstetricians about ACS was not investigated and may present an opportunity for improvement.

## In conclusion

The overall incidence of ACS use among Chinese level III maternal hospitals in our cohort was 71.2%. The incidence of prenatal ACS use was highest among preterm infants who were 28–31 weeks GA and in pregnancies with medical complications. There are opportunities for improving ACS use in Chinese hospitals.

## Data Availability

The data used and/or analyzed during the current study are available from the corresponding author on reasonable request.

## Abbreviations

ACS: Antenatal corticosteroids
NRDS: Neonatal respiratory distress syndrome
NEC: Necrotizing enterocolitis
IVH: Intraventricular hemorrhage
REIN-EPIQ: REduction of Infection in Neonatal intensive care units using the Evidence-based Practice for Improving Quality
NICU: Neonatal intensive care unit
HDP: Hypertensive disorders of pregnancy
PROM: Prelabor rupture of membranes
TRIPS: Transport risk index of physiologic stability
BPD: Bronchopulmonary dysplasia
PVL: Periventricular leukomalacia
EOS: Early-onset sepsis
ROP: Retinopathy of prematurity
CS: Cesarean section
SGA: Small for gestational age
